# What waiver? A qualitative exploration of awareness of the X-waiver among underserved patients and providers and the impact on tele-OUD services uptake

**DOI:** 10.3389/fpsyt.2025.1589546

**Published:** 2025-07-16

**Authors:** Omolola Adepoju, Laura De La Roche, Carlos G. Fuentes, Ryan Wong, Lauren Gilbert

**Affiliations:** ^1^ Humana Institute, Tilman J. Fertitta Family College of Medicine, University of Houston, Houston, TX, United States; ^2^ Department of Health Systems and Population Health Sciences, Tilman J. Fertitta Family College of Medicine, University of Houston, Houston, TX, United States; ^3^ Department of Health Systems and Population Health Sciences, University of Houston, Houston, TX, United States

**Keywords:** opioid use disorder (OUD), qualitative research, X-waiver, telemedicine, opioid use disorder treatment services

## Abstract

**Introduction:**

The X-waiver is a federal requirement that previously mandated healthcare providers to obtain special certification to prescribe buprenorphine for OUD. The removal of the X-Waiver represents a significant shift in U.S. drug policy aimed at expanding access to evidence-based treatment. Comparing the waiver *vs*. post-waiver era for telemedicine-related opioid use disorder services (TOUDS) can yield critical insights into access, utilization, and patient outcomes, particularly in underserved populations. However, a significant gap remains in the literature exploring the qualitative perspective of both providers and underserved minoritized patients. This study, part of a longitudinal study, compares provider and patient perceptions of TOUDS access in the waiver and post-waiver era.

**Methods:**

Participants were recruited from a local OUD treatment clinic in Houston TX. We conducted in-depth qualitative interviews on eight participants, comprising of health care providers and self-identified Black and Hispanic patients. Interviews were completed in the post-X-waiver era and asked about their TOUDS experiences. Data was analyzed using reflexive thematic analysis.

**Results:**

Data analysis generated three themes: Understanding and Navigating Telemedicine for OUD treatment; Characterizing the Impacts of Distinct Forms of Stigma on TOUDS; and Waiver Awareness. Findings highlighted the benefits of TOUDS as well as barriers to uptake. Participants advocated for targeted efforts to increase public knowledge among patients and providers. They described the negative impact of stigma originating from their communities and providers. Finally, results highlighted a lack of awareness surrounding the x-waiver although, providers articulated an increase in colleague inquiries regarding how to navigate the provision of TOUDS.

**Discussion:**

TOUDS were viewed more positively by providers and patients in the post-waiver era; however, this change was not attributed to the removal of the wavier. Specifically, many patient-identified barriers prior to the removal of the X-waiver were not reported in our post-X-waiver study, and TOUDS benefits were instead emphasized. Our findings provide critical insight into the perceptions of TOUDS from both providers and patients in the post-X-waiver era that can direct policies on awareness and access.

## Introduction

The use of virtual modalities, including telemedicine, to address access to care gaps has gained popularity over the past few years. During this same period, the field of Opioid Use Disorder (OUD) research has explored how OUD treatments can benefit from Telemedicine’s rising popularity. Earlier work suggests that telemedicine has the potential to reduce stigma associated with seeking OUD treatment by providing greater privacy (1). However, telemedicine also presents challenges, particularly for minoritized groups facing the digital divide—defined as limited access to technology, low digital literacy, and lack of reliable internet (2). Telemedicine initiatives for OUD treatment in predominantly black communities across cities like Washington DC (3), Baltimore (4), San Francisco (5), and Philadelphia (6) underscore the importance of tailored interventions to address technology barriers in racial and ethnic sub-populations. Although early studies during the pandemic showed promise, further research is needed in a post-pandemic context to understand the sustained impact of telemedicine on OUD treatment access.

Until recently, reimbursement, privacy, and licensing regulations limited the use of OUD-related telemedicine (7). However, the passage of the CARES Act during the COVID-19 pandemic, coupled with the Drug Enforcement Administration announcement suspending the in-person visit requirement for controlled substance prescriptions, as outlined in the Ryan Haight Online Pharmacy Consumer Protection Act, led to a significant shift (8, 9). Many states subsequently relaxed licensure requirements for telemedicine providers (7). In 2023, Congress furthered efforts to enhance access to OUD care by eliminating the waiver requirement that required practitioners to submit a Notice of Intent to prescribe medications like buprenorphine (10). These regulatory changes aimed to broaden access to medication-assisted treatment (MAT), particularly through telemedicine, thereby improving continuity of care and health outcomes for individuals living with OUD.

The removal of the X-waiver, a federal requirement that previously mandated healthcare providers to obtain special certification to prescribe buprenorphine for OUD, represents a significant shift in U.S. drug policy aimed at expanding access to evidence-based treatment. Historically, the X-waiver, established under the Drug Addiction Treatment Act of 2000 (11), limited the number of clinicians authorized to prescribe buprenorphine, a medication proven to reduce opioid-related morbidity and mortality. Its removal under the Mainstreaming Addiction Treatment Act of 2022 (12) aligns with public health efforts to integrate OUD treatment into general medical practice, reducing stigma and increasing accessibility. By allowing any prescriber with a DEA license to prescribe buprenorphine without additional training or restrictions, this policy change is expected to improve treatment availability (13, 14), particularly in underserved and rural areas where opioid-related deaths are disproportionately high. While concerns remain about the need for adequate clinician education on addiction management, the policy is widely regarded as a crucial step in addressing the opioid crisis by normalizing and expanding access to MAT (13, 14).

In our previous research on telemedicine access during the X-waiver era, research participants reported experiencing less social anxiety and intrusion into their personal lives with telemedicine, as they are not required to physically attend a clinic where they may be recognized by others (15). However, cultural influences and perceptions of trust and confidentiality significantly impacted participants’ willingness to engage with telemedicine services. This was repeatedly reported among marginalized populations historically subjected to discrimination and mistreatment in public sectors such as healthcare and law enforcement (16). Other participants highlighted concerns about privacy, with some fearing unauthorized recording of telemedicine conversations, and potential legal implications if law enforcement accesses their telemedicine records, or the possibility of friends or family overhearing their conversations related to OUD treatment (17–19).

In this study, we sought to understand patients’ and providers’ perception of the impact of the removal of the X-waiver on telemedicine-related OUD access. Studies of this nature provide insight regarding the continued benefits and/or barriers to TOUDS use, which can guide future modifications and subsequent delivery of TOUDS.

## Methods

### Participants

Participants were recruited from a local substance use treatment clinic and included five patients (three female, two male) diagnosed with OUD who had received treatment, as well as three health care providers. Participants were eligible for participation if they were at least 18-years old, were able to understand and speak in English, identified as being either Black of Hispanic, and were either receiving or providing treatment for OUD. Participants self-identified as Black or African American, with an average age of 35.6 (SD =10.44). Among the patients, three reported having been prescribed buprenorphine (Suboxone), and two reported having been prescribed methadone as part of their medication-assisted treatment. Participants were compensated with $50 gift cards for their participation.

### Procedure

Individual interviews took between 15–63 minutes to complete (M=41.73), and were conducted by authors LG and CF, both with expertise in qualitative research involving individuals living with OUD disorders. All interviews were conducted online using a professional university Zoom account and the auto-transcription function was employed. Transcripts were subsequently double-checked and formatted by trained researcher assistants prior to analysis. Ethical approval for this study was granted by the Institutional Review Board (IRB) at the University of Houston (IRB# 00004276).

### Measures

A semi-structured interview guide was established based on existing literature and feedback from the Community Research Advisory Board from the University of Houston Research Center in Minority Institution. The questions were open-ended and structured to understand previous experiences related to opioid use disorder, telemedicine, and the x-waiver. Specifically, patients were asked about their history with addiction, use of related resources, experiences and perceptions of TOUDS, and the knowledge and understanding of the x-waiver and the impact of its removal. The provider interview followed a similar structure but excluded questions about personal opioid use history and telemedicine usage, instead emphasizing their comfort with TOUDS.

### Data analysis

Reflexive thematic analysis was employed to analyze transcripts of both the patients and providers. We followed Braun and Clarke’s (2021) recommendations pertaining to reflexive thematic analysis. Specifically, we adhered to their six-phase process, including engaging with, reviewing, and analyzing the data prior to generating and reporting the findings. Initial review of the data determined that all perspectives were better analyzed inclusively, rather than reviewing the patients’ perspectives separately from the providers. We maintained an inductive approach through data analysis to accurately capture the full picture of the utility of TOUDS (Thorne et al., 1997; Lincoln & Guba, 1985).

### Trustworthiness

Following recommendations within the qualitative field, we employed numerous forms of trustworthiness to increase the rigor of our study and the generated findings (Lincoln & Guba, 1982; 1985; Stahl & King, 2020). We integrated three methods of trustworthiness: researcher triangulation (Stahl & King, 2020); persistent observation, and reflexivity (Berger, 2015; Korstjens & Morser, 2018). Throughout the analytical process, the researchers met on a biweekly basis to discuss the data and associated generated codes, subthemes, and themes. This was completed to ensure different perspectives from researchers across disciplines were considered and potential biases mitigated. Further, during data collection and analysis, persistent observation of the data was conducted, and numerous meetings to discuss the data as it was analyzed occurred. Reflexivity involved researchers actively reflecting on their own experiences and preconceptions both individually and via group discussions to acknowledge any researcher bias and limit its influence on data interpretation.

## Results

**Figure 1:**
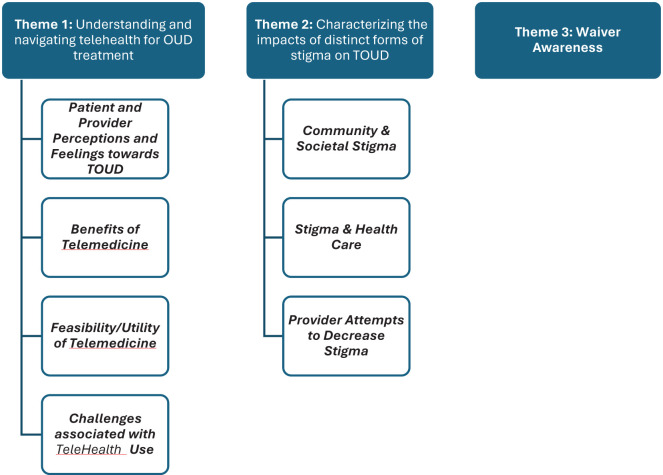
Findings organized by themes and subthemes.

Data was coded using descriptive coding, in which researchers reviewed and reflected on the data prior to assigning a representative code. Line-by-line coding was completed to ensure all data from all transcripts was included in the data analysis and represented within the generated findings. Codes were subsequently reviewed and organized into categories that accurately represented the breadth and depth of the data. Finally, categories were organized into the generated subthemes and themes reported in the findings. Data analysis generated three primary themes: Understanding and Navigating Telemedicine for OUD treatment; Characterizing the Impacts of Distinct Forms of Stigma on TOUDS; and Waiver Awareness (**Figure 1**). Subthemes are organized within relevant themes. Direct quotes are integrated throughout the results and represent verbatim dialogue from participant interviews.

### Theme 1: Understanding and navigating telemedicine for OUD treatment

Theme one was broken down into four subthemes that included discussions focused on patient and provider perceptions and experiences of TOUDS:

#### Patient and provider perceptions and feelings towards TOUDS

Participants were asked, and identified, their awareness of, and perceptions towards, TOUDS. Varied history of use was reported across patients, ranging from current use of TOUDS that provided recovery medication, to limited familiarity and no previous TOUDS engagement. Patients discussed how they did not perceive TOUDS to be a generally known option within their community (i.e., among others living with addiction). However, once TOUDS was described, patients were excited and felt TOUDS should be better publicized to individuals who may benefit from it. Unfortunately, while the providers included in this study were accepting of TOUDS, with some providing such care, they felt that generally, TOUDS was not positively perceived or supported by other providers. For example, one provider discussed the limited bandwidth of health care practitioners, and staff turnover, while another discussed how the impact of integrating an additional form of care (i.e., TOUDS) to their plate was perceived as a potential barrier to continued use due to the additional time required.

#### Benefits of telemedicine

Patients articulated their preference for virtual care when possible and those who had previously used TOUDS provided positive reviews regarding their experience and decreased travel requirements. These patients highlighted TOUDS as especially beneficial for short appointments (e.g., prescription renewal). Patients liked that they “don’t have to drive 20 minutes there 20 minutes back, that’s a 40-minute trip just for 10 minutes” (Patient 3). TOUDS further mitigated location eligibility requirements as patients described not obtaining previous care because they were not eligible due to their location.

The benefits related to childcare were further discussed. Patients noted that TOUDS allowed them to complete sessions at home and therefore eliminated the need to find childcare assistance. One patient advocated for the need to only be seen in-person once a month to receive recovery-related medications, explaining that “while participating in in-person resources providing recovery medication, they want to see you every week to test your levels, which gets discouraging [because] missing an appointment means you can’t get your medication, TOUDS has allowed me have once-a-month in person visits” (patient 5).

Patients using TOUDS further described an established and trusted rapport with their health care provider that supported their recovery journey. In discussing rapport, some patients felt heard and respected by the health care professionals they engaged with virtually. Furthermore, some patients felt that TOUDS may be beneficial supporting initial help-seeking behaviors for those starting their recovery journey. Specifically, due to the ease and immediacy of care provision being “just a call away” (Patient 1), one patient reported that TOUDS could be the key to getting individuals living with addiction to take the first step towards recovery. The uncertainty associated with the initial steps of seeking help was therefore perceived to be mitigated by the ease of TOUDS. In contrast, providers were concerned that some patients incorrectly think that telemedicine means immediate same-day appointments - this is not always realistic or feasible.

Similar to patients, providers recognized the benefits of TOUDS and mitigation of barriers, recognizing the same barriers to accessing care as patients, and discussed how they tried to alleviate them where possible. For example, Provider 2 described how they “provide free services, which include counselling and because of the location where we are, and just to make it accessible to our patients, we provide our sessions through Zoom”. Providers further felt that TOUDS alleviated many barriers to accessing in-person OUD care because “it [TOUDS] has really taken down a lot of barriers for some individuals who may not be able to get into treatment, like residential treatment or detox [.] and being able to connect individuals with the medication [and] providers” (Provider 3).

TOUDS was further identified as a possible opportunity to alleviate general mistrust associated with healthcare providers and systems. Mistrust in healthcare providers and services was an identified barrier to care uptake by both patients and providers. Providers recognized that

“most of them [patients living with addiction] really do not trust the healthcare system so expecting to physically show up in a clinic and kind of be subjected to a lot of the stigma that they’ve received over their lives is difficult for them. So, giving them an option where they can do it from their phone is just a lot more comfortable for many of them, which means more of them are likely to reach out for help” (Provider 1).

In sum, challenges associated with recovering from addiction were recognized by providers, and they were adamant at trying to make the process as easy and comforting as possible for patients. These challenges were further identified by patients accessing TOUDS, who described telehealth as decreasing the experienced emotional distress and anxiety surrounding accessing addiction care. Patients described how accessing care virtually was perceived to mitigate the potential for being stigmatized for receiving recovery-related care. For these reasons, some patients identified that virtual care was their mode of preference, as opposed to in-person care.

#### Feasibility/utility of telemedicine

Providers perceived the resistance to TOUDS by other providers to be, in part, due to the reliance and perceived necessity of urine samples/screeners for patients being prescribed and provided recovery medications. However, providers questioned the necessity of these screening tests; some providers felt they were not entirely necessary given relapse is an understood part of the recovery process and medications should not be withheld if a relapse occurs. Furthermore, false positive drug tests occur, and providers were concerned that falsely telling a patient that they received a positive drug screening could do significant damage to their recovery, such as causing a relapse. Providers therefore “don’t see why they couldn’t [provide OUD care via telehealth] you know, this is not a diagnosis or treatment that relies on a physical exam much so this is something that really is quite doable over telehealth” (Provider 1). Providers further described how urine screens can be made more accessible to supplement TOUDS. Specifically, urine screens could be completed through partner agencies with convenient locations for patients who can then ship samples to provider locations for testing. This method allows primarily virtual care to be utilized, while urine screens remain a component that becomes more accessible and convenient for patients.

Patients felt accessing TOUDS to be simple and the necessity for an electronic device capable of videoconferences feasible via their cellular device. Some patients made note of how some aspects of in-person may not be possible in virtual care, such as non-verbal communication when video was not employed. Further, if a physical exam was necessary for recovery care, then patients may prefer in-person appointments as the medical professional may identify medical concerns that the patient is unaware of.

#### Challenges associated with TeleHealth use

While TOUDS was generally positively reviewed by patients and providers, barriers to its use were identified. Specifically, while patients said they had the necessary technology to access TOUDS, they recognized others may not. Patients further discussed challenges associated with stable internet, which was also highlighted by providers. Specifically, providers recalled challenges with patients obtaining and maintaining steady internet connections during TOUDS sessions, which, while manageable, they felt was a barrier to engagement. Providers were further concerned about the technological literacy of some patients. One provider noted “for the older generation, it’s often just technology. They’re not as savvy with apps or even understanding what an app is” (Provider 1). However, methods to combat these challenges were identified, as Provider 1 further explained that “we usually have [peer recovery] coaches call them, walk them through the process, send them an email, and guide them step by step”. One patient who had not previously used TOUDS expressed concern regarding the quality of care they may receive via telemedicine. The ability for patients to build and establish a relationship and mutual trust with their provider was a salient concern among patients, with telemedicine-related automation prompts highlighted as frustrating.

Providers further outlined some challenges associated with ensuring confidentiality with TOUDS due to patients wanting to complete their virtual appointments with others (e.g., family members) in the same room. These requests required providers to complete waivers regarding the sharing of information for patients wanting to complete sessions wherein others could overhear their discussions. Furthermore, providers found themselves having to articulate the importance of individual sessions to family members who were reluctant for the patient to meet with the provider alone. While these concerns were raised by a provider, they further noted that the barriers were worthwhile to overcome, as their patients were unlikely to meet at all if in-person sessions were required.

Finally, patients identified the current stage of recovery as a barrier to TOUDS engagement. Specifically, one patient noted that TOUDS should only be considered a realistic option to patients when they are in a frame of mind or time in their recovery journey where they are receptive to help.

### Theme 2: Characterizing the impacts of distinct forms of stigma on TOUDS

Theme two included discussions related to stigma associated with addiction and recovery. Participants described different types of experienced and perceived stigma associated with obtaining addiction care. Further, methods that were intended or perceived to decrease stigma are discussed.

#### Community & societal stigma

Participants were concerned about societal stigma targeting individuals living with addiction. Community stigma was identified, such that stigma surrounding addiction and associated help-seeking were taboo. Patient 1 stated simply that

“I am an African American woman [ … ] it’s more so our community, it’s more so my own people that are negative [ … ] In the Black community [ … ] certain things are looked down upon, like counseling, showing emotions, [ … ] going to treatment, addiction”.

Stigma from within the Black community was further described by Patient 3 who said that members of the Black community living with addiction “suffer for longer because of the stigmas [ … ] around it”. While patients said they worked to prevent stigma from negatively affecting them or their behaviors, they felt that many people do not admit to needing help or experiencing addiction related challenges due to fear of being stigmatized.

Providers expressed similar concerns regarding community support. They described scenarios in which their patient’s sobriety was not taken seriously, and that the chronic nature of recovery was not fully understood by community members. This concerned providers, who recognized recovery is a life-long process, stating that “nobody’s ever discharged from our program” (Provider 3).

Patients further described stigma from within the recovery community;

“I call them [ … ] the little drug gangs. You got the cocaine gangs *Oh I’ll never do, I would never do heroin!* You got the heroin people *oh man, you smoke crack – I will never do that!* (Patient 3).

Inter-recovery stigma was further recognized by providers. This was described as stigma surrounding the use of medication to support recovery such that individuals in recovery that utilized medication to support their recovery were stigmatized by those who did not use medication. Recognition of societal stigma was further discussed by Provider 1, who stated that

“of course, there’s kind of the generalized stigma as well in society, [ … ] movies and TV [ … ] we see folks who look really dirty often or just thieves and [ … ] just get this really bad rap [ … ] [then when] there’s a town meeting and they’re gonna put a Methadone clinic on our street and everyone’s [ … ] upset about because they think *oh everyone’s houses is gonna get broken into*. So unfortunately, these folks [individuals living with addiction] are really painted as villains”.

#### Stigma & health care

Providers described how “the majority of patients I see with opioid use disorder began their use with a prescription” (Provider 1) which instigated their continued distrust of healthcare professionals. Patients explained that they initially trusted their medical providers when taking prescribed medication which resulted in their OUD, but subsequently became skeptical of future advice and/or prescriptions from the same providers. Once they brought up challenges or concerns associated with their prescribed medications, they were labeled as addicts and not provided further care, which led to feelings of betrayal in addition to mistrust.

While some participants described positive interactions and being respected by health care providers, both providers and patients felt that patients receiving or seeking out OUD care often experienced stigma by medical professionals, which negatively impacted their recovery and perception of OUD care. Provider 3 stated “I have seen it and patients have told us that as well, that’s why some patients won’t go to the hospital because they feel like [ … ] stigmatized or that the medical providers think [ … ] they’re drug seeking”. Patients further described experienced and anticipated stigma from emergency medicine providers. Negative experiences with health care providers were perceived by patients to be more prevalent when seeking out recovery medication as opposed to behavioral health services. Providers described how they’ve “had pharmacies tell me they don’t stock medication because they don’t want those types of patients around” (Provider 1). Stigma surrounding OUD therefore extended to various health-care services. However, provider stigma was perceived to not be as prevalent in providers of TOUDS compared to in-person services.

#### Provider attempts to decrease stigma

Providers identified a number of methods they practiced to put their patients at ease. They recognized that many individuals living with addiction have negative past experiences with health care professionals and associated judgment from them. Therefore, they consciously made adjustments to their presentation;

“I typically don’t wear a white coat; I feel like that’s one thing [that] just visually can be kind of like I’m stuffy and come from this [ … ] background where people judge you a lot. So, I usually just kind of wear scrubs or, you know, business casual” (Provider 1).

Provider 1 further described how they tried to create a safe non-judgmental environment and patient-provider relationship through intentional language and discussion. For example, Provider 2 noted that “instead of saying things like dirty or clean you know – was your urine drug screen results as expected or unexpected?”. Therefore, an intentional use of language to reframe addiction from a moral failure to a chronic medical condition was employed by providers to decrease any subtle stigmatization associated with patient use of addiction-related resources. Providers further indicated they consciously work to remove discriminating labels, such as addict, from discussions with patients.

### Theme 3: Waiver awareness

There was limited awareness of the waiver, or associated changes to services and service delivery in the post-waiver era. Patients were unaware of the waiver or what it represented, and further unaware that requirements surrounding the waiver had recently been removed. Similarly, some providers were unaware of the wavier removal, as one provider stated, “I actually didn’t know that the waiver was removed to be honest” (Provider 2). While providers who were aware of the waiver removal perceived the changes in waiver requirements to be beneficial, they did not initially identify salient changes to services they, or the organization in which they worked, provided. Importantly, the providers included in this sample noted that their roles predominantly included the provision of counseling services and therefore they did not prescribe any medications. Therefore, while they felt that the removal of the waiver requirement was positive, it was not perceived to have impacted their day-to-day procedures or provision of care. They further did not feel as though it had impacted the providers they worked with who prescribed recovery medication.

However, providers did identify changes in the behaviors of outside or new colleagues. Specifically, they reported receiving more inquiries regarding their experience with providing OUD care, and the logistics therein, by colleagues since requirements surrounding the waiver had changed. Furthermore, providers “feel there has been a shift there that there’s a lot more support” (Provider 3) in the post-era waiver in which more organizations are willing to support OUD recovery.

## Discussion

This study sought to qualitatively investigate both provider and patient perspectives of TOUDS in the post-waiver era. While telehealth is a growing area of research across topics, few researchers to date have sought to understand the experiences and perspectives of patients and clinicians to obtain a full-picture understanding of TOUDS uptake in the wake of the elimination of the waiver requirement and associated relaxation of legal restrictions on care provision. The qualitative nature of our analysis enabled both benefits and barriers surrounding TOUDS to be identified and described; these will be discussed as they relate to, and extend, existing literature. Our findings extend previous research that identifies TOUDS as a viable means to enhance accessibility and decrease stigma in OUD care, and highlights aspects of TOUDS to promote increased access and uptake.

A central theme was the impact of stigma on TOUDS uptake, with patients perceiving less stigma when using TOUDS compared to in-person treatment. Our findings further support previous research, which suggests the provision of virtual treatment options may decrease perceived stigma exposure and in turn increase patients access and uptake of recovery services. Specifically, our findings align with Couch et al. (2024), who found that patients identified concerns regarding both public stigma and privacy concerns, were potentially mitigated by the “social distance of telehealth” (20).

Previous work by Weiner et al. (2024) found that clinicians acknowledged benefits surrounding patient accessibility and flexibility, which were also identified by providers in our sample. Weiner et al. (2024) further reported concerns from clinicians regarding the need for preserved patient-clinician rapport and cautioning against using TOUDS as a blanket solution without considering unique patient barriers (e.g. technology, affordability, counseling) to improved care (21). Of note, some clinicians reported that a subset of their patients preferred in-person care as a means of facilitating rapport, further highlighting the potential role of TOUDS as a complement to in person care (21–24). Additionally, Wyte-Lake et al. (2024) found that clinicians noted greater control over scheduling for telehealth visits with patients stable in recovery, but cautioned against completely transitioning over to telehealth care, preferring to establish rapport with new patients in-person and gradually shifting to telehealth care for routine stable visits (15). This aligns in part with our findings, in which some providers indicated that the balance between TOUDS and in-person OUD treatment was highly dependent on the needs of the patient, and therefore on a case-by-case basis. That is, barriers to TOUDS uptake from other clinicians was perceived to stem from feasibility in workload bandwidth, familiarity, and willingness by providers in our sample, in addition to general awareness of the ability to do so following the removal of the X-waiver. While participants in our sample did not indicate a preference between telephone or video visits, Wyte-Lake et al. (2025) found that patients typically preferred telephone to video visits if given the option within telehealth (15).

The removal of the X-waiver, along with its training requirements, aimed to expand buprenorphine access and reduce barriers to care by allowing more providers to prescribe recovery medications. However, to date, studies have not demonstrated significant associations between changes in training or waiver requirements and improved access to care (25–27).Prior to the removal of the x-wavier, researchers found TOUDS to be a viable alternative to in-person care, highlighting high long-term retention rates controlling for geography and ethnicity, and comparable uptake to in-person care (28, 29) Specifically, Nguyen et al. (2023) found that TOUDS that initiated and provided buprenorphine for recovery resulted in lower overdose rates and higher patient engagement rates compared to in-person care (30). Couch et al. (2024) reported patients valued the increased control over their treatment setting and flexibility, which fostered positive perceptions of clinician trust when using TOUDS (20). Similarly, Weiner et al. (2024) found that patients associated TOUDS with increased flexibility, adding that patients felt OUD could be treated without the need for in-person requirements, which imposed significant undesirable travel, work, and childcare burdens on patients (21). Wyte-Lake et al. (2024) further supported these findings, noting that patients and clinicians unanimously recognized telehealth as improving care accessibility through reduced travel, work, and psychological burdens, enhancing continuous OUD care (15). These results directly align with our findings, indicting strong support by patient populations for the integration and provision of TOUDS to support recovery. Specifically, the benefits highlighted in our findings, including the flexibility in scheduling, mitigation of travel requirements and associated costs, and accessibility of care, align and extend the findings in the literature.

## Limitations

While methods of trustworthiness were integrated to increase the rigor of our study, there are limitations that must be recognized. It is important to note that due to our sample size, we cannot assure that the perspectives of our interviewed patients and clinicians are representative. Furthermore, we could not completely minimize confounding factors related to COVID-19 influencing patients’ and providers’ perspectives on TOUDS in the post-waiver era, as this pandemic’s duration in the US closely overlapped with the elimination of the X-waiver. Notwithstanding, our findings provide a novel understanding of perspectives of TOUDS in the post-waiver era.

## Conclusions

Patients and providers found TOUDS to be a valuable asset to their OUD care and recovery following elimination of the X-waiver. Patients emphasized benefits to TOUDS in flexibility and access to care, notably opting for telehealth appointments whenever possible. However, clinicians continued to caution against overstretching the feasibility of telehealth without adequate community support, and patients broadly saw continuing limited awareness of telehealth as a drawback to wider adoption. Our findings provide critical insight into opportunities for patients and clinicians to partner in making TOUDS scalable and sustainable in the post-X-waiver era.

## Data Availability

The datasets presented in this article are not readily available because the nature of the collected interview data prohibit the sharing of data to ensure participant confidentiality. Requests to access the datasets should be directed to oadepoju@central.uh.edu.
